# Association of anthropometric measures and cardiovascular risk factors in children and adolescents: Findings from the Aboriginal Birth Cohort study

**DOI:** 10.1371/journal.pone.0199280

**Published:** 2018-06-21

**Authors:** Angela Gialamas, Angela Kinnell, Murthy N. Mittinty, Belinda Davison, Gurmeet Singh, John Lynch

**Affiliations:** 1 School of Public Health, University of Adelaide, Adelaide, Australia; 2 Menzies School of Health Research, Charles Darwin University, Darwin, Northern Territory, Australia; 3 School of Social and Community Medicine, University of Bristol, Bristol, United Kingdom; Shanghai Institute of Hypertension, CHINA

## Abstract

This study examined the association of anthropometric measures including height, leg length, trunk length and body mass index (BMI) at 11 and 18 years with systolic blood pressure (SBP), diastolic blood pressure (DBP), total cholesterol, low-density lipoprotein cholesterol (LDL-c) and high-density lipoprotein cholesterol (HDL-c) at 11 and 18 years. We analysed data from 661 participants from the Aboriginal Birth Cohort study–a longitudinal study based in the Northern Territory, Australia. Associations between anthropometric measures and cardiovascular risk factors were investigated in linear regression analyses adjusted for confounding, with imputation for missing data. In adjusted analyses, increasing leg length [males: 0.47mmHg/cm (0.23, 0.72); females: 0.50mmHg/cm (0.18, 0.83)], trunk length [males: 0.50mmHg/cm (0.28, 0.73); females: 0.57mmHg/cm (0.33, 0.81)] and height [males: 0.32mmHg/cm (0.16, 0.48); females: 0.32mmHg/cm (0.12, 0.52)] at 11 years was associated with higher SBP at 11 years. When these exposures were measured at 18 years the effect on SBP at 18 years had attenuated, and only increased trunk length was associated with higher SBP at 18 years for both sexes [males: 0.46mmHg/cm (0.05, 0.87); females: 0.69mmHg/cm (0.30, 1.08)]. We observed little association between height, leg length and trunk length and DBP, total cholesterol, LDL-c and HDL-c. Increased BMI was associated with elevated SBP and DBP at 11 and 18 years. Our findings suggest that height, leg length, and trunk length measured at 11 and 18 years was generally not associated with cardiovascular risk factors at 11 and 18 years. However, greater childhood BMI was associated with higher blood pressure and this association persisted into adolescence. This study contributes to the limited body of evidence on the association between measures of early anthropometry and cardiovascular risk among the Australian Aboriginal population.

## Introduction

Numerous studies in western countries have found that greater adult height and, in particular longer legs, has been associated with lower pulse pressure, systolic blood pressure, and decreased risk for cardiovascular disease (CVD) and death [1–6]. However, evidence that childhood height and its components (leg length and trunk length) are associated with adult outcomes has been less clear. For instance, analyses of deaths occurring up to 1995 from the Boyd Orr cohort (n = 2990) found that greater childhood height and leg length was associated with decreasing risk of cardiovascular mortality [7]. In contrast, a later study of the Boyd Orr cohort (n = 2642) that included more death data than the previous publication, found no association between childhood height, leg length and trunk length with all cause, all cardiovascular, ischaemic heart disease (IHD),stroke mortality and self-reported IHD [8].

While a substantial body of research exists on the effects of small size at birth with later CVD and death [9–12], few studies have evaluated other measures of postnatal and childhood growth, such as height and the association with cardiovascular risk factors earlier in life. Unlike studies measuring CVD and its risk factors in adults, greater height in childhood has been found to be associated with higher blood pressure in childhood, [13, 14] and that trunk length rather than leg length was associated with raised blood pressure [14]. For example, in an American cohort of socioeconomically advantaged children (n = 1153 at age 3; n = 1086 at age 8) longer leg length, adjusted for trunk length, was not associated with blood pressure at ages 3 and 8 [14].

Growth trajectories in children may vary by ethnicity, and be affected by the quality of the living environment during the years of physical development [15]. Children who are raised in households at the poorer end of the socioeconomic spectrum are also at higher risk of poorer cardiovascular outcomes [1]. Notwithstanding recent improvements, Australia’s Aboriginal peoples, when compared with other Australians are more likely to experience poorer health, socioeconomic, and nutritional deprivation across the life course, and are at higher risk of prematurely dying from CVD [16]. Undertaking research to understand the effect of childhood anthropometry on cardiovascular risk is important as it may enable early identification of risk and intervention, therefore reducing the avoidable burden of adult ill health and premature mortality.

Using data from the Aboriginal Birth Cohort study (ABC study), the aim of the present study was to investigate whether anthropometric measurements including birth weight, birth length, height, leg length, trunk length and body mass index at 11 and 18 years was associated with measures of cardiovascular risk at 11 and 18 years. To our knowledge no previous study has examined associations between aspects of anthropometry and CVD risk factors in early life among Aboriginal peoples, whose childhoods were mainly spent in rural and remote regions of Northern Australia.

## Materials and methods

### Study design and sample

The ABC study is a longitudinal examination of a birth cohort of Aboriginal Australians. Study design and sample information for the ABC study has been described elsewhere [17]. In brief, infants were eligible for enrolment in the study if they were live born singletons delivered at the Royal Darwin Hospital, Northern Territory, between January 1987 and March 1990 to a mother recorded as Aboriginal in the delivery suite register [17]. A total of 686 infants were recruited into the study (response rate 55.4% of an eligible 1238 infants). The number of Aboriginal live births for the Northern Territory population in 1987–1990 was approximately 3503 births [18], thus the 686 infants recruited into the study represented a reasonably large proportion of a small population base. In line with available research funding, subsequent follow-up of this cohort has occurred at a mean age of 11 years between December 1998 and January 2001, and 18 years between January 2006 and February 2008. Our analyses use the first three waves of data collected at birth, 11 and 18 years. Informed consent was provided by participants in the ABC study at each time point. At study recruitment the Aboriginal research assistant explained the proposed study to prospective participants. If agreement was obtained, the neonatal paediatrician visited the Aboriginal mothers, discussed the aims of the study and obtained verbal maternal permission to recruit the baby into the cohort [17]. Informed written consent was obtained by the parent/guardian at the 11 year follow-up and from the participant at the 18 year follow-up. Ethics approval was obtained prior to each follow-up from the Human Research Ethics Committee of Northern Territory Department of Health and the Menzies School of Health Research, including the Aboriginal Ethical Sub-committee which has the power of veto.

### Anthropometry measures

The main exposure variables included birth weight, birth length, leg length, trunk length, leg-to-trunk ratio, height, and body mass index. Birth weights were recorded to the nearest gram using a balance scale, crown-heel lengths were measured with a length board using standard anthropometric methods [17, 19]. At the 11 and 18 year follow-up anthropometric measurements were assessed by a trained researcher using standardised techniques [20]. Participants were measured in light clothing and barefoot. Height was measured to the nearest millimetre with a portable stadiometer (ADA MZ10017) which was calibrated daily, on changing location or as required. Trunk length was assessed by sitting height (participant sitting upright with hips at a right angle and thighs parallel to the floor) minus box height (a flat sitting surface parallel to the floor, which enabled flush contact with the wall generating a right angle between the sitting platform and the wall). Leg length was calculated as the difference between standing height and trunk length. Leg-to-trunk ratio was calculated by dividing leg length by trunk length, a measure that has also been used in previous studies. Weight was measured to the last complete 0.1 kilogram with a digital electronic scale (TBF-521, Tanita Corporation Illinois, USA). Body mass index (BMI) was calculated by dividing weight (kilograms) by height (metre) squared. BMIs were converted to sex- and age-specific z scores using the World Health Organization’s reference data for child growth [21, 22] and the zanthro program for Stata.

### Cardiovascular risk factors

The outcomes measured at the 11 and 18 year follow-up included systolic blood pressure (SBP), diastolic blood pressure (DBP), total cholesterol, low-density lipoprotein cholesterol (LDL-c) and high-density lipoprotein cholesterol (HDL-c). Blood pressure was obtained three times, on the right arm, whilst sitting, and post resting using an automatic oscillatory unit (Lifesigns BP Monitor, Welch Allyn, New York, USA). Mid upper arm circumference was measured at both times and the appropriate cuff size was used. Blood samples were collected, and participants were defined as fasting if they had fasted for 8 hours or more prior to the sample collection (330 and 453 were defined as fasting at the 11 year and 18 year follow-up respectively). Blood samples were centrifuged post clotting as soon as possible, within two hours after collection, frozen, and transported in cold-boxes to the laboratory. Cholesterol concentrations were measured enzymatically (11 year follow-up using the Hitachi 917 auto analyser, Roche, Switzerland and at 18 year follow-up using the colorimetric analyser, Roche Modular).

### Confounding

Potential confounding factors included: child age; gestational age; birth weight for gestational age z score [23]; place of residence at birth, 11 and 18 years; pubertal status; smoking and alcohol use at 18 years. The place of residence at birth, 11 and 18 years was classified into three broad geographic regions: urban; remote/very remote; or other areas. The neonatal paediatrician completed gestational age assessment within 4 days of birth using the Dubowitz Scoring System.[19] Pubertal growth results in an increase in height as well as an increase in blood pressure hence pubertal status was included as a potential confounding factor [24]. Pubertal staging was assessed at age 11 by a paediatrician according to Tanner staging [25, 26].

### Statistical analysis

Linear regression models were used to estimate associations between anthropometric measures at birth, 11 and 18 years and cardiovascular risk factors at 11 and 18 years. Three sequential models were used to estimate the effect of anthropometric measures on outcomes. The first model included only age. The second model added place of residence, birth length, birth weight for gestational age z score, and gestational age. When examining associations between leg length or trunk length on outcomes the third model included all variables in models 1 and 2, and the other component of height (leg or trunk length). The decision to adjust for the other component of height was based on previous studies [4, 14, 27] and allowed us to investigate the independent contributions of each. Analyses at age 11 adjusted for pubertal status in addition to the above factors. Analyses at age 18 also adjusted for smoking and alcohol use. Results are presented for males and females separately, because of the different growth patterns between boys and girls [28] and the potential sex differences in cardio-metabolic risk [29]. All analyses were carried out in Stata SE 14 (TX, USA).

### Analytic sample

**Fig 1:**
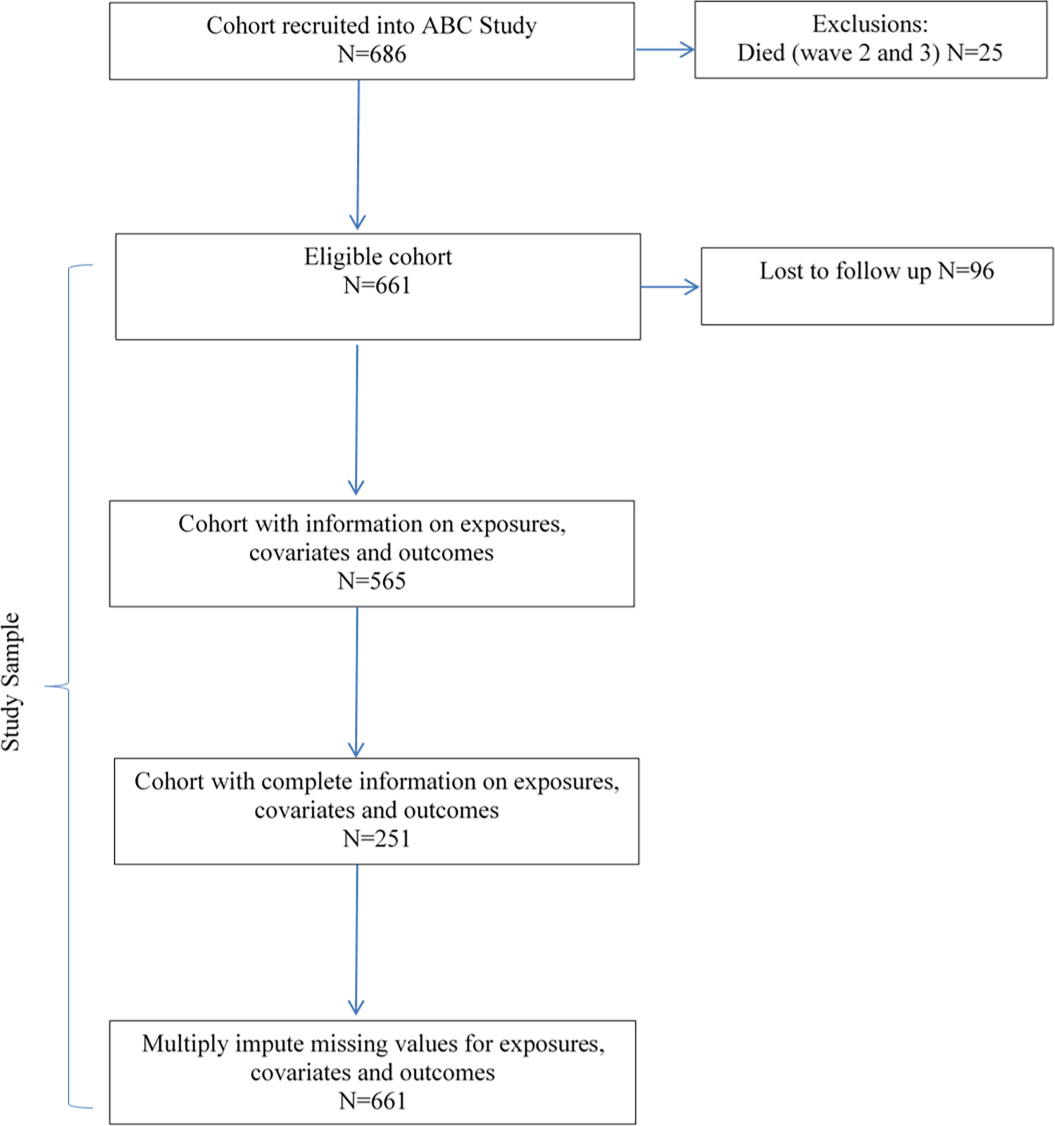
Eligible cohort and numbers included for analyses.

A total of 661 children (347 males; 314 females) were included in the analysis. Fig 1 shows that of the 686 infants recruited at birth, 25 were known to have died by the 18 year follow-up (n = 19 at wave 2; n = 6 at wave 3), and therefore these children were excluded from the analysis. Of the remaining 661 children, 410 (62.0%) had missing data in one or more variables of interest leaving 251 children with complete information. To minimise non-response bias, multiple imputation by chained equations was used to impute missing values [30]. Imputed datasets were generated under the missing at random assumption that uses observed variables in the dataset to predict missingness and estimate parameters [31]. In accordance with best practice [32] the variables used to predict missingness in the imputation model included the exposures, outcomes, and confounders. Twenty imputed datasets were generated and the results of the imputed analyses were combined using Rubin’s rules [33]. Results using the complete-case data were not substantively different from the imputed analysis and would not change the conclusions of this study; therefore, we report the imputed results (results for participants with complete-case data are shown in S1–S4 Tables).

## Results

Characteristics of the study participants at birth, at the childhood and adolescent visit are shown in Table 1 (results for the response sample are shown in S5 Table). The mean age of children at the childhood visit was 11.4 years (range, 8.8–14.0 years) and 18.3 years (range, 15.5–21.2 years) at the adolescent visit. At 11 years SBP was similar for both sexes (107.8 vs 107.6 mmHg), but by age 18 males had higher SBP than females (113.2 vs 107.1 mmHg). DBP, cholesterol, LDL-c and HDL-c was similar for both sexes at 11 and 18 years. Mean WHO BMI z-scores were -0.66 (S.D. 1.6) and -0.55 (S.D. 1.6) for males and -0.40 (S.D. 1.5) and -0.24 (S.D 1.5) for females at 11 and 18 years respectively.

**Table 1:** Characteristics of ABC study participants for the multiply imputed sample.

A			
	Total (n = 661)	Males (n = 347)	Females (n = 314)
**Birth**			
Birth length (cm), mean (SD)	48.8 (2.9)	49.3 (3.0)	48.2 (2.8)
Birth weight (kilograms), mean (SD)	3.04 (0.63)	3.13 (0.64)	2.95 (0.59)
Gestational age (weeks), mean (SD)	38.3 (1.7)	38.4 (1.7)	38.2 (1.7)
Place of residence, n (%)			
Urban	138 (20.9)	76 (21.9)	62 (19.8)
Remote/very remote	465 (70.3)	242 (69.7)	223 (71.0)
Other areas	58 (8.8)	29 (8.4)	29 (9.2)
**Childhood visit (~11 years)**			
Age at wave 2 (years), mean (SD)	11.4 (1.1)	11.6 (1.1)	11.3 (1.1)
Place of residence, n (%)			
Urban	142 (21.5)	79 (22.8)	63 (20.1)
Remote/very remote	448 (67.8)	230 (66.3)	218 (69.4)
Other areas	71 (10.7)	38 (10.9)	33 (10.5)
Pubertal status, n (%)			
Pre-pubertal	332 (50.2)	227 (65.4)	105 (33.4)
Pubertal	329 (49.8)	120 (34.6)	209 (66.6)
Height (cm), mean (SD)	145.2 (10.5)	145.1 (10.4)	145.3 (11.2)
Leg length (cm), mean (SD)	72.0 (6.4)	72.1 (6.7)	72.0 (6.0)
Trunk length (cm), mean (SD)	72.4 (6.5)	72.1 (6.7)	72.7 (6.7)
Weight (kg), mean (SD)	36.6 (13.6)	35.6 (12.6)	37.8 (14.7)
BMI z score (WHO growth chart)	-0.53 (1.5)	-0.66 (1.6)	-0.40 (1.5)
Total cholesterol, mmol/L, mean (SD)	4.0 (0.7)	4.1 (0.7)	3.9 (0.7)
HDL-c, mmol/L, mean (SD)	1.2 (0.3)	1.2 (0.3)	1.1 (0.2)
LDL-c, mmol/L, mean (SD)	2.2 (0.6)	2.3 (0.7)	2.2 (0.6)
Systolic blood pressure (mmHg), mean (SD)	107.7 (10.2)	107.8 (9.9)	107.6 (10.6)
Diastolic blood pressure (mmHg), mean (SD)	68.0 (7.1)	67.4 (7.0)	68.7 (7.2)
**Adolescent visit (~18 years)**			
Age at wave 3 (years), mean (SD)	18.3 (1.0)	18.4 (1.0)	18.2 (1.1)
Place of residence, n (%)			
Urban	187 (28.3)	109 (31.4)	78 (24.8)
Remote/very remote	385 (58.3)	189 (54.5)	196 (62.4)
Other areas	89 (13.5)	49 (14.1)	40 (12.7)
Do you smoke tobacco/cigarettes, n (%)			
No	188 (28.4)	92 (26.5)	96 (30.6)
Used to smoke	10 (1.5)	8 (2.3)	2 (0.6)
Smoke sometimes	463 (70.1)	247 (71.2)	216 (68.8)
Alcohol use, n (%)			
Never or only tried it once	332 (50.2)	145 (41.8)	187 (59.6)
Used to use it but not anymore	23 (3.5)	12 (3.5)	11 (3.5)
Sometimes	306 (46.3)	190 (54.7)	116 (36.9)
Height (cm), mean (SD)	168.5 (8.7)	174.5 (6.9)	161.8 (6.0)
Leg length (cm), mean (SD)	83.9 (5.7)	87.3 (4.5)	80.1 (4.4)
Trunk length (cm), mean (SD)	84.4 (4.8)	86.6 (4.6)	82.1 (4.1)
Weight (kg), mean (SD)	60.0 (19.0)	62.8 (19.4)	56.9 (18.2)
B			
BMI z score (WHO growth chart)	-0.41 (1.7)	-0.55 (1.6)	-0.24 (1.5)
Total cholesterol, mmol/L, mean (SD)	4.0 (0.8)	4.1 (0.8)	4.0 (0.8)
HDL-c, mmol/L, mean (SD)	1.0 (0.2)	1.0 (0.2)	1.0 (0.2)
LDL-c, mmol/L, mean (SD)	2.3 (0.7)	2.4 (0.7)	2.3 (0.7)
Systolic blood pressure (mmHg), mean (SD)	110.3 (11.8)	113.2 (11.3)	107.1 (11.2)
Diastolic blood pressure (mmHg), mean (SD)	69.0 (7.8)	69.2 (0.1)	68.5 (7.7)

Tables 2–4 present regression coefficients from a range of models assessing associations of anthropometric measures at birth, childhood and adolescence with SBP and DBP at the childhood and adolescent visit for males and females. Estimates are for increments in SBP (mmHg), DBP (mmHg), cholesterol (mmol/L), LDL-c (mmol/L), HDL-c (mmol/L) for every 1 cm increment in birth weight, birth length, height or its components.

**Table 2:** Associations between anthropometric measures at birth and childhood with systolic and diastolic blood pressure measured at the childhood and adolescent visit for males using the multiply imputed data.

Exposure	Model	Systolic Blood Pressure (mmHg) at childhood visit		Systolic Blood Pressure (mmHg) at adolescent visit		Diastolic Blood Pressure (mmHg) at childhood visit		Diastolic Blood Pressure (mmHg) at adolescent visit	
		β (95% CI)	P	β (95% CI)	P	β (95% CI)	P	β (95% CI)	P
**MALES**									
**Birth**									
Birth weight (kg)	1	0.39 (-1.37, 2.15)	0.66	1.11 (-1.18, 3.40)	0.33	0.34 (-0.91, 1.60)	0.59	0.36 (-1.13, 1.87)	0.62
Birth length (cm)	1	-0.02 (-0.40,0.34)	0.87	0.27 (-0.20, 0.74)	0.26	-0.01 (-0.27, 0.24)	0.92	0.11 (-0.20, 0.42)	0.48
**Childhood**									
Height (cm)	1	0.28 (0.14, 0.41)	0.00	0.27 (0.11, 0.43)	0.00	0.07 (-0.02, 0.16)	0.13	0.14 (0.03, 0.25)	0.01
	2	0.32 (0.16, 0.48)	0.00	0.18 (0.01, 0.35)	0.03	0.09 (-0.01, 0.20)	0.09	0.09 (-0.02, 0.21)	0.11
Leg length (cm)	1	0.28 (0.06, 0.50)	0.01	0.42 (0.15, 0.68)	0.00	0.14 (-0.01, 0.31)	0.07	0.19 (-0.00, 0.39)	0.06
	2	0.31 (0.07, 0.54)	0.01	0.44 (0.19, 0.69)	0.00	0.16 (-0.01, 0.34)	0.08	0.44 (0.19, 0.69)	0.00
	3	0.47 (0.23, 0.72)	0.00	0.45 (0.20, 0.70)	0.00	0.20 (0.01, 0.40)	0.04	0.45 (0.20, 0.70)	0.00
Trunk length (cm)	1	0.38 (0.18, 0.58)	0.00	0.20 (-0.04, 0.44)	0.10	0.06 (-0.07, 0.20)	0.35	0.13 (-0.01, 0.29)	0.08
	2	0.37 (0.14, 0.61)	0.00	0.12 (-0.15, 0.39)	0.39	0.07 (-0.09, 0.23)	0.39	0.01 (-0.15, 0.19)	0.83
	3	0.50 (0.28, 0.73)	0.00	0.24 (-0.02, 0.50)	0.07	0.12 (-0.04, 0.29)	0.14	0.06 (-0.09, 0.23)	0.42
Leg-to-trunk ratio	1	-3.72 (-14.4,7.02)	0.49	7.37 (-5.55,20.31)	0.25	2.81 (-4.41,10.05)	0.44	2.32 (-6.78,11.43)	0.61
BMI WHO z scores	1	1.74 (0.98, 2.50)	0.00	1.66 (0.84, 2.47)	0.00	0.61 (0.04, 1.18)	0.04	0.85 (0.21, 1.48)	0.01
	2	1.89 (1.05, 2.73)	0.00	1.43 (0.54, 2.33)	0.00	0.71 (0.09, 1.33)	0.02	0.71 (0.01, 1.41)	0.04

**Model 1:** age

**Model 2:** age, place of residence, birth length, birth weight for gestational age z score, gestational age and pubertal status (pubertal status adjusted only in childhood visit)

**Model 3:** age, place of residence, birth length, birth weight for gestational age z score, gestational age, pubertal status (only adjusted in childhood visit), other component of current height (leg length for trunk length and vice versa

**Table 3:** Associations between anthropometric measures at birth and childhood with systolic and diastolic blood pressure measured at the childhood and adolescent visit for females using the multiply imputed data.

Exposure	Model	Systolic Blood Pressure (mmHg) at childhood visit		Systolic Blood Pressure (mmHg) at adolescent visit		Diastolic Blood Pressure (mmHg) at childhood visit		Diastolic Blood Pressure (mmHg) at adolescent visit	
		β (95% CI)	P	β (95% CI)	P	β (95% CI)	P	β (95% CI)	P
**FEMALES**									
**Birth**									
Birth weight(kg)	1	-0.34 (-2.54,1.84)	0.75	0.35 (-2.15, 2.86)	0.78	-0.70 (-2.30, 0.89)	0.38	0.26 (-1.41, 1.93)	0.75
Birth length (cm)	1	-0.11 (-0.58,0.34)	0.61	0.04 (-0.50, 0.58)	0.87	-0.14 (-0.48, 0.19)	0.39	0.04 (-0.31, 0.40)	0.81
**Childhood**									
Height (cm)	1	0.35 (0.17, 0.52)	0.00	0.21 (0.04, 0.39)	0.01	0.11 (-0.01, 0.23)	0.08	0.09 (-0.02, 0.21)	0.10
	2	0.32 (0.12, 0.52)	0.00	0.15 (-0.02, 0.33)	0.09	0.08 (-0.06, 0.23)	0.25	0.05 (-0.09, 0.20)	0.45
Leg length (cm)	1	0.48 (0.18, 0.78)	0.02	0.29 (-0.01, 0.60)	0.05	0.22 (0.01, 0.43)	0.04	0.11 (-0.09, 0.31)	0.28
	2	0.42 (0.08, 0.76)	0.01	0.44 (0.19, 0.69)	0.00	0.19 (-0.03, 0.43)	0.09	0.32 (0.03, 0.61)	0.02
	3	0.50 (0.18, 0.83)	0.00	0.30 (0.01, 0.59)	0.04	0.22 (-0.01, 0.45)	0.06	0.30 0.01, 0.59)	0.04
Trunk length (cm)	1	0.56 (0.35, 0.78)	0.00	0.29 (0.04, 0.54)	0.02	0.17 (0.01, 0.33)	0.03	0.18 (0.01, 0.36)	0.03
	2	0.50 (0.24, 0.76)	0.00	0.18 (-0.09, 0.45)	0.18	0.12 (-0.06, 0.30)	0.19	0.14 (-0.03, 0.32)	0.12
	3	0.57 (0.33, 0.81)	0.00	0.18 (-0.14, 0.51)	0.26	0.15 (-0.03, 0.33)	0.11	0.15 (-0.07, 0.37)	0.19
Leg-to-trunk ratio	1	-4.90 (-16.8, 7.0)	0.41	0.01 (-14.3,14.3)	0.99	1.21 (-6.86, 9.28)	0.76	-2.72 (-11.9, 6.53)	0.56
BMI WHO z scores	1	1.93 (1.04, 2.82)	0.00	1.40 (0.40, 2.39)	0.00	0.56 (-0.04, 1.16)	0.07	0.72 (0.09, 1.35)	0.02
	2	1.74 (0.76, 2.73)	0.00	1.09 (0.06, 2.12)	0.03	0.45 (-0.23, 1.14)	0.19	0.59 (-0.10, 1.29)	0.09

Model 1: age

**Model 2:** age, place of residence, birth length, birth weight for gestational age z score, gestational age and pubertal status (pubertal status adjusted only in childhood visit)

**Model 3:** age, place of residence, birth length, birth weight for gestational age z score, gestational age, pubertal status (only adjusted in childhood visit), other component of current height (leg length for trunk length and vice versa)

**Table 4:** Associations between anthropometric measures at adolescence with systolic and diastolic blood pressure measured at the adolescent visit for males and females using the multiply imputed data.

Exposure	Model	Systolic Blood Pressure (mmHg) at adolescent visit		Diastolic Blood Pressure (mmHg) at adolescent visit	
		β (95% CI)	P	β (95% CI)	P
**MALES**					
Height (cm)	1	0.27 (0.08, 0.47)	0.01	0.09 (-0.04, 0.23)	0.15
	2	0.16 (-0.06, 0.39)	0.16	0.01 (-0.15, 0.17)	0.88
Leg length (cm)	1	0.05 (-0.29, 0.40)	0.75	-0.04 (-0.29, 0.20)	0.72
Trunk length (cm)	1	0.63 (0.32, 0.95)	0.00	0.25 (0.04, 0.47)	0.02
	2	0.46 (0.05, 0.87)	0.02	0.13 (-0.13, 0.40)	0.32
	3	0.46 (0.05, 0.87)	0.02	0.13 (-0.13, 0.40)	0.33
Leg-to-trunk ratio	1	-25.7 (-47.9,-3.4)	0.02	-13.5 (-28.8,1.69)	0.08
	2	-15.9 (-40.10, 8.23)	0.19	-9.32 (-25.69, 7.04)	0.25
BMI WHO z scores	1	1.70 (0.90, 2.51)	0.00	0.94 (0.38, 1.51)	0.00
	2	1.53 (0.59, 2.48)	0.00	0.85 (0.23, 1.48)	0.001
**FEMALES**					
Height (cm)	1	0.24 (0.01, 0.49)	0.04	0.09 (-0.06, 0.26)	0.24
	2	0.20 (-0.05, 0.46)	0.11	0.07 (-0.11, 0.25)	0.44
Leg length (cm)	1	-0.07 (-0.40, 0.26)	0.67	-0.14 (-0.36, 0.07)	0.20
Trunk length (cm)	1	0.78 (0.43, 1.13)	0.00	0.43 (0.18, 0.68)	0.00
	2	0.69 (0.29, 1.08)	0.00	0.39 (0.10, 0.68)	0.01
	3	0.69 (0.30, 1.08)	0.00	0.38 (0.09, 0.67)	0.01
Leg-to-trunk ratio	1	-30.8 (-50.3, -11.4)	0.00	-17.6 (-31.61, -3.74)	0.01
	2	-22.4 (-43.02, -1.83)	0.03	-17.1 (-30.9,-3.37)	0.02
BMI WHO z scores	1	1.68 (0.67, 2.68)	0.00	1.00 (0.35, 1.64)	0.00
	2	1.49 (0.43, 2.55)	0.00	0.98 (0.24,1.65)	0.00

**Model 1:** age

**Model 2:** age, place of residence, birth length, birth weight for gestational age z score, gestational age, smoking, and alcohol use

**Model 3:** age, place of residence, birth length, birth weight for gestational age z score, gestational age, smoking, alcohol use, and other component of current height (leg length for trunk length and vice versa)

Birth weight or birth length was not associated with SBP or DBP at 11 or 18 years. In fully adjusted models, increasing leg length [males: 0.47mmHg/cm (0.23, 0.72; females: 0.50mmHg/cm (0.18, 0.83)], trunk length [males: 0.50mmHg/cm (0.28, 0.73); females: (0.57mmHg/cm (0.33, 0.81)] and height [males: 0.32mmHg/cm (0.16, 0.48; females: (0.32mmHg/cm (0.12, 0.52)] at 11 years was associated with higher levels of SBP at 11 years. Leg length at 11 years was also associated with higher levels of SBP and DBP at 18 years for males and for females, whereas increasing height was only associated with higher SBP for males at 18 years (Tables 2 and 3).

In adjusted analyses, leg length and height measured at 18 years was not associated with SBP at 18 years (Table 4). In contrast, increased trunk length at 18 years was associated with higher SBP at 18 years for males (0.46mmHg/cm (0.05, 0.87)) and females (0.69mmHg/cm (0.29, 1.08)). We observed little consistent association between height, leg length and trunk length with DBP.

BMI at 11 years was positively associated with SBP at 11 and 18 years for both males and females (Tables 2 and 3). BMI measured at 18 years maintained its positive association with SBP and DBP at 18 years of age for both sexes (Table 4).

S6 and S7 Tables present the models of birth weight, birth length, height, leg length, trunk length, leg-to-trunk ratio and BMI at 11 and 18 years with total cholesterol, LDL-c and HDL-c at 11 and 18 years for males and females. All of these analyses were conducted for the sample as a whole. In sum, we found little association between these anthropometric measures at 11 and 18 years and total cholesterol, LDL-c or HDL-c at 11 and 18 years. To address whether results might differ between fasting and non-fasting individuals for total cholesterol, LDL-c and HDL-c, sensitivity analyses were performed. Similar results were obtained when examining fasting and non-fasting individuals separately.

## Discussion

Our study adds to the literature in this area by examining associations between aspects of early life anthropometry and CVD risk factors in an Aboriginal birth cohort whose childhoods were largely spent in rural and remote areas of Northern Australia. We find little evidence to support the hypothesis that aspects of growth at early ages including birth weight, birth length, height, leg length, and trunk length measured at 11 and 18 years was consistently associated with cardiovascular risk factors such as SBP, DBP, total cholesterol, LDL-c and HDL-c at 11 and 18 years. Our study demonstrated that increasing leg length, trunk length, and height measured at 11 years was associated with higher SBP at 11 years in both sexes, but when these exposures were measured at 18 years the effect on SBP at 18 years had attenuated, and only increased trunk length measured at 18 was associated with higher SBP at 18 years for males, and SBP and DBP for females. However, greater childhood BMI was associated with higher SBP and DBP for males and females, and this association persisted into adolescence.

Our finding that trunk length at 11 years was positively associated with SBP at 11 years supports results obtained by Regnault et al [14] who found a positive association between trunk length and SBP at 3 and 8 years. However, our findings also differ from those obtained by Regnault et al [14] who found no association of leg length for a given trunk length. This discrepancy in results may be due to differences between the ages of children and populations studied. Our study included older children, of whom 49% had commenced puberty at the childhood visit, from Aboriginal families who experience higher rates of socioeconomic disadvantage, infant under-nutrition and infection, and low birth weight [34], as compared to the socioeconomically advantaged, pre-pubertal, American children followed up by Regnault et al [14]. On the other hand the difference in results between studies may reflect the view that the association between components of height and blood pressure change after puberty [14].

Our findings also differ from Kouda et al [35] who studied 3219 children aged 10–11 years, in Iwata City in Shizuoka prefecture, Japan, who found an inverse association between height and total cholesterol in both male and females. In our study, we only found an inverse association between leg length measured at 18 and cholesterol at 18 years for males. One explanation for the discrepancy in findings could be due to differences in growth patterns between Aboriginal and Japanese children. The Aboriginal children were, on average, taller (males: 145.1cm vs. 138.0cm; females: 145.3cm vs. 139.1cm), heavier (males: 35.7kg vs. 33.4kg; females: 37.8kg vs. 33.6kg) and had lower cholesterol levels (males: 4.1 mmol/L vs 4.5 mmol/L; females: 3.9 mmol/L vs. 4.5 mmol/L) at ~11 years than the Japanese children.

The developmental origins hypothesis proposes that early life experiences and conditions including poor social, economic and nutritional conditions during pregnancy and early childhood can negatively influence early growth and development and contribute to later morbidity and mortality [36–39]. The effects of characteristics such as low birth weight have been shown in studies to be associated with CVD and its biological risk factors [40]. However, in the present study we found no association between birth weight or birth length with cardiovascular risk factors including SBP, DBP, total cholesterol, LDL-c and HDL-c at 11 and 18 years. Our findings are in line with a recent Mendelian randomisation study that found little support for an effect of birth weight on risk of IHD [41]. Furthermore, other measurements of early childhood growth including height, leg length, and trunk length were not consistently associated with SBP, DBP, cholesterol, LDL-c and HDL-c in childhood or adolescence. This does not mean associations may not appear at older ages, but they are not evident in this population by age 18.

A positive association between BMI, SBP and DBP in childhood and adolescence was found in our study. These findings are consistent with those from other studies in the general Australian population and other developed countries that have demonstrated a positive association between BMI and blood pressure [42–44]. Only one other study has investigated the relationship between BMI and blood pressure in Australian Aboriginal children. Results of our study are in line with findings from the prospective cohort study (SEARCH) of urban Aboriginal children (0–17 years) in New South Wales that found child BMI positively associated with diastolic and systolic blood pressure [45].

The children in our study were not representative of all Aboriginal children in Australia, most of whom live in large urban centres [46]. However, compared with nationally representative data, the height and weight distributions of 11 year old males and females were similar to what we found in our study (average height males:145.1cm (ABC study) vs. 141.3cm (national data); females:145.3cm vs. 146.5cm; average weight males: 35.6kg vs. 36.1kg; females: 37.8kg vs. 41.1kg) [47]. Compared to self-reported height and weight statistics collected in the 2005 Australian National Health Survey for a sample of 18–24 year old Aboriginal adolescents, the Aboriginal adolescents in our study were on average shorter (males:174.5cm vs. 180cm; females:161.8cm vs. 165.5cm) and weighed less (males:62.8kg vs. 78.4kg; females:56.9kg vs. 63.7kg), [48] but this discrepancy may be due to the inclusion of older adults in the national health survey.

Several limitations need to be acknowledged. First, our study was limited by a small sample that may have reduced our power to detect small associations. Nevertheless, the number recruited and retained in this study is large, particularly as Australia’s Aboriginal population represents a small proportion of the total population (1.5% of the total population,1986 Census) [46]. Second, only 50–68.5% of children at the childhood and adolescent visit fasted eight or more hours before their lipid pathology test. However, fasting samples are not essential for evaluation of cardiovascular risk, and non-fasting lipid profiles have been recommended for use in the majority of patients, including children [49]. Third, our analyses were not able to adjust for potential confounding factors such as family income that influence a child’s growth and cardiovascular risk. Although we could not adjust for income our use of the variable “place of residence” was considered a good proxy for the child’s socioeconomic circumstances. Fourth, blood pressure was measured using an automated oscillatory unit. Concerns have been raised regarding the accuracy of using oscillometric blood pressure devices compared to the gold standard, namely the manual mercury sphygmomanometer in children [50]. However, as all blood pressure measurements were performed in the same way for all participants, using the same device, any measurement errors are unlikely to have introduced bias into the study, as there is no reason to believe that the error would be differential with regard to the outcome.

## Conclusion

We found that birth weight, birth length, height, leg length, and trunk length measured at 11 and 18 years was generally not associated with cardiovascular risk factors at 11 and 18 years among an Aboriginal population. However, increased BMI was consistently associated with increased SBP and DBP in both sexes. This study contributes to the limited body of evidence on the association between measures of early anthropometry and cardiovascular risk among the Australian Aboriginal population.

## Supporting information

S1 Table Associations between anthropometric measures at birth and childhood with systolic and diastolic blood pressure measured at the childhood and adolescent visit for males and females using the complete-case data. (DOCX)

S2 Table Associations between anthropometric measures at adolescence with systolic and diastolic blood pressure measured at the adolescent visit for males and females using the complete-case data. (DOCX)

S3 Table Associations between anthropometric measures at birth and childhood with cholesterol, HDL-C, LDL-c measured at the childhood and adolescent visit for males and females using the complete-case data. (DOCX)

S4 Table Associations between anthropometric measures at adolescence with cholesterol, HDL-C, LDL-C, measured at the adolescent visit for males and females using the complete-case data. (DOCX)

S5 Table Characteristics of ABC study participants–number who responded to each variable. (DOCX)

S6 Table Associations between anthropometric measures at birth and childhood with cholesterol, HDL-c and LDL-c measured at the childhood and adolescent visit for males and females using the multiply imputed data. (DOCX)

S7 Table Associations between anthropometric measures at adolescence with cholesterol, HDL-c and LDL-c measured at the adolescent visit for males and females using the multiply imputed data. (DOCX)
