# Supplementary material for: Association of anthropometric measures and cardiovascular risk factors in children and adolescents: Findings from the Aboriginal Birth Cohort study
Source: PLoS One. 2018 Jun 21;13(6):e0199280. doi: 10.1371/journal.pone.0199280 (PMC6013209; doi:10.1371/journal.pone.0199280)
Supplement: S1 Table — (DOCX) [file pone.0199280.s001.docx]

Supplementary Table 1: Associations between anthropometric measures at birth and childhood with systolic and diastolic blood pressure measured at the childhood and adolescent visit for males and females using the complete-case data

| Exposure | Model | Systolic Blood Pressure (mmHg)  at childhood visit | | | Systolic Blood Pressure (mmHg)  at adolescent visit | | | Diastolic Blood Pressure (mmHg)  at childhood visit | | | Diastolic Blood Pressure (mmHg)  at adolescent visit | | |
| --- | --- | --- | --- | --- | --- | --- | --- | --- | --- | --- | --- | --- | --- |
|  |  | n | β (95% CI) | P | n | β (95% CI) | P | n | β (95% CI) | P | n | β (95% CI) | P |
| **MALES** |  |  |  |  |  |  |  |  |  |  |  |  |  |
| **Birth** |  |  |  |  |  |  |  |  |  |  |  |  |  |
| Birth weight (kg) | 1 | 292 | 0.36 (-1.38, 2.11) | 0.68 | 223 | 1.84 (-0.47, 4.15) | 0.11 | 292 | 0.45 (-0.78, 1.69) | 0.46 | 223 | 0.55 (-1.05, 2.16) | 0.49 |
| Birth length (cm) | 1 | 287 | -0.08 (-0.47,0.30) | 0.67 | 220 | 0.34 (-0.13, 0.82) | 0.16 | 287 | -0.07 (-0.34,0.20) | 0.60 | 220 | 0.10 (-0.23, 0.43) | 0.53 |
| **Childhood** |  |  |  |  |  |  |  |  |  |  |  |  |  |
| Height (cm) | 1 | 291 | 0.37 (0.23, 0.51) | 0.00 | 212 | 0.37 (0.20, 0.54) | 0.00 | 291 | 0.09 (-0.01, 0.19) | 0.07 | 212 | 0.22 (0.09, 0.34) | 0.00 |
|  | 2 | 233 | 0.51 (0.32, 0.70) | 0.00 | 180 | 0.41 (0.20, 0.62) | 0.00 | 233 | 0.17 (0.03, 0.30) | 0.01 | 180 | 0.22 (0.07, 0.37) | 0.00 |
| Leg length (cm) | 1 | 222 | 0.18 (-0.04, 0.42) | 0.11 | 166 | 0.50 (0.21, 0.78) | 0.00 | 222 | 0.10 (-0.05, 0.27) | 0.19 | 166 | 0.31 (0.11, 0.51) | 0.00 |
|  | 2 | 181 | 0.51 (0.21, 0.80) | 0.00 | 140 | 0.56 (0.25, 0.87) | 0.00 | 181 | 0.29 (0.08, 0.49) | 0.01 | 140 | 0.30 (0.07, 0.53) | 0.01 |
|  | 3 | 181 | 0.60 (0.31, 0.89) | 0.00 | 140 | 0.58 (0.28, 0.89) | 0.00 | 181 | 0.31 (0.10, 0.52) | 0.00 | 140 | 0.31 (0.08, 0.54) | 0.01 |
| Trunk length (cm) | 1 | 223 | 0.38 (0.18, 0.58) | 0.00 | 167 | 0.37 (0.08, 0.65) | 0.01 | 223 | 0.06 (-0.07, 0.21) | 0.35 | 167 | 0.23 (0.03, 0.44) | 0.02 |
|  | 2 | 182 | 0.44 (0.17, 0.72) | 0.00 | 141 | 0.29 (-0.02, 0.61) | 0.07 | 182 | 0.09 (-0.09, 0.28) | 0.33 | 141 | 0.12 (-0.10, 0.36) | 0.27 |
|  | 3 | 181 | 0.52 (0.25, 0.79) | 0.00 | 140 | 0.34 (0.04, 0.65) | 0.03 | 181 | 0.13 (-0.05, 0.32) | 0.15 | 140 | 0.15 (-0.07, 0.38) | 0.18 |
| Leg-to-trunk ratio | 1 | 222 | -5.73 (-16.35,4.89) | 0.28 | 166 | 7.64 (-6.14, 21.4) | 0.27 | 222 | 1.90 (-5.45, 9.26) | 0.61 | 166 | 4.99 (-4.77, 14.7) | 0.31 |
| BMI WHO z scores | 1 | 291 | 2.41 (1.74, 3.09) | 0.00 | 212 | 2.21 (1.29, 3.14) | 0.00 | 291 | 0.82 (0.31, 1.34) | 0.00 | 212 | 1.14 (0.48, 1.80) | 0.00 |
|  | 2 | 233 | 2.86 (2.02, 3.69) | 0.00 | 180 | 2.04 (0.93, 3.14) | 0.00 | 233 | 1.12 (0.50, 1.74) | 0.00 | 180 | 1.22 (0.41, 2.02) | 0.00 |

| Exposure | Model | Systolic Blood Pressure (mmHg)  at childhood visit | | | Systolic Blood Pressure (mmHg)  at adolescent visit | | | Diastolic Blood Pressure (mmHg)  at childhood visit | | | Diastolic Blood Pressure (mmHg)  at adolescent visit | | |
| --- | --- | --- | --- | --- | --- | --- | --- | --- | --- | --- | --- | --- | --- |
|  |  | n | β (95% CI) | P | n | β (95% CI) | P | n | β (95% CI) | P | n | β (95% CI) | P |
| **FEMALES** |  |  |  |  |  |  |  |  |  |  |  |  |  |
| **Birth** |  |  |  |  |  |  |  |  |  |  |  |  |  |
| Birth weight (kg) | 1 | 261 | 0.10 (-2.10, 2.31) | 0.92 | 227 | 0.14 (-2.34, 2.63) | 0.90 | 261 | -0.67 (-2.18, 0.84) | 0.38 | 227 | 0.19 (-1.47, 1.86) | 0.82 |
| Birth length (cm) | 1 | 256 | 0.01 (-0.47, 0.49) | 0.95 | 222 | 0.02 (-0.52, 0.57) | 0.92 | 256 | -0.11 (-0.44, 0.21) | 0.48 | 222 | 0.04 (-0.32, 0.41) | 0.80 |
| **Childhood** |  |  |  |  |  |  |  |  |  |  |  |  |  |
| Height (cm) | 1 | 261 | 0.56 (0.41, 0.71) | 0.00 | 214 | 0.25 (0.07, 0.42) | 0.01 | 261 | 0.19 (0.07, 0.30) | 0.00 | 214 | 0.12 (0.00, 0.23) | 0.05 |
|  | 2 | 228 | 0.61 (0.41, 0.82) | 0.00 | 195 | 0.26 (0.07, 0.46) | 0.01 | 228 | 0.21 (0.06, 0.36) | 0.01 | 195 | 0.15 (0.02, 0.28) | 0.02 |
| Leg length (cm) | 1 | 197 | 0.43 (0.14, 0.72) | 0.00 | 168 | 0.18 (-0.13, 0.51) | 0.25 | 197 | 0.14 (-0.05, 0.35) | 0.14 | 168 | 0.00 (-0.22, 0.23) | 0.99 |
|  | 2 | 171 | 0.35 (0.01, 0.70) | 0.04 | 152 | 0.27 (-0.07, 0.62) | 0.12 | 171 | 0.15 (-0.07, 0.39) | 0.18 | 152 | 0.06 (-0.17, 0.30) | 0.61 |
|  | 3 | 171 | 0.47 (0.13, 0.80) | 0.01 | 152 | 0.24 (-0.10, 0.60) | 0.16 | 171 | 0.19 (-0.04, 0.42) | 0.11 | 152 | 0.03 (-0.20, 0.27) | 0.78 |
| Trunk length (cm) | 1 | 197 | 0.59 (0.33, 0.85) | 0.00 | 168 | 0.22 (-0.08, 0.54) | 0.14 | 197 | 0.17 (-0.01, 0.35) | 0.06 | 168 | 0.17 (-0.04, 0.38) | 0.12 |
|  | 2 | 171 | 0.58 (0.26, 0.90) | 0.00 | 152 | 0.18 (-0.15, 0.53) | 0.27 | 171 | 0.15 (-0.07, 0.37) | 0.16 | 152 | 0.17 (-0.05, 0.40) | 0.14 |
|  | 3 | 171 | 0.66 (0.34, 0.97) | 0.00 | 152 | 0.15 (-0.19, 0.49) | 0.38 | 171 | 0.18 (-0.04, 0.40) | 0.10 | 152 | 0.17 (-0.06, 0.40) | 0.15 |
| Leg-to-trunk ratio | 1 | 197 | -6.78 (-19.3,5.80) | 0.28 | 168 | -1.68(-17.7, 14.3) | 0.83 | 197 | -0.68 (-9.20, 7.83) | 0.87 | 168 | -6.46 (-17.4, 4.5) | 0.24 |
| BMI WHO z scores | 1 | 261 | 3.24 (2.38, 4.09) | 0.00 | 214 | 2.09 (0.98, 3.19) | 0.00 | 261 | 1.00 (0.36, 1.63) | 0.00 | 214 | 1.18 (0.42, 1.94) | 0.00 |
|  | 2 | 228 | 3.77 (2.71, 4.83) | 0.00 | 195 | 2.13 (0.89, 3.37) | 0.00 | 228 | 1.34 (0.55, 2.13) | 0.00 | 195 | 1.31 (0.47, 2.15) | 0.00 |

**Model 1:** age

**Model 2:** age, place of residence, birth length, birth weight for gestational age z score, gestational age and pubertal status (pubertal status adjusted only in childhood visit)

**Model 3:** age, place of residence, birth length, birth weight for gestational age z score, gestational age, pubertal status (only adjusted in childhood visit), other component of current height (leg length for trunk length and vice versa)
