# Supplementary material for: Association of anthropometric measures and cardiovascular risk factors in children and adolescents: Findings from the Aboriginal Birth Cohort study
Source: PLoS One. 2018 Jun 21;13(6):e0199280. doi: 10.1371/journal.pone.0199280 (PMC6013209; doi:10.1371/journal.pone.0199280)
Supplement: S2 Table — (DOCX) [file pone.0199280.s002.docx]

Supplementary Table 2: Associations between anthropometric measures at adolescence with systolic and diastolic blood pressure measured at the adolescent visit for males and females using the complete-case data

| Exposure | Model | Systolic Blood Pressure (mmHg)  at adolescent visit | | | Diastolic Blood Pressure (mmHg)  at adolescent visit | | |
| --- | --- | --- | --- | --- | --- | --- | --- |
|  |  | **n** | **β (95% CI)** | **P** | **n** | **β (95% CI)** | **P** |
| **MALES** |  |  |  |  |  |  |  |
| Height (cm) | 1 | 222 | 0.35 (0.13, 0.57) | 0.00 | 222 | 0.11 (-0.03, 0.26) | 0.13 |
|  | 2 | 166 | 0.46 (0.16, 0.75) | 0.00 | 166 | 0.18 (-0.02, 0.40) | 0.09 |
| Leg length (cm) | 1 | 216 | 0.13 (-0.20, 0.47) | 0.42 | 216 | 0.02 (-0.21, 0.25) | 0.88 |
| Trunk length (cm) | 1 | 216 | 0.64 (0.31, 0.96) | 0.00 | 216 | 0.24 (0.01, 0.47) | 0.03 |
|  | 2 | 163 | 0.51 (0.05, 0.97) | 0.02 | 163 | 0.20 (-0.13, 0.53) | 0.24 |
|  | 3 | 163 | 0.49 (0.04, 0.95) | 0.03 | 163 | 0.19 (-0.14, 0.52) | 0.25 |
| Leg-to-trunk ratio | 1 | 216 | -23.55 (-45.1, -1.9) | 0.03 | 216 | 0.24 (0.01, 0.47) | 0.04 |
|  | 2 | 163 | 3.86 (-22.97, 30.69) | 0.77 | 163 | 3.03 (-16.3, 22.3) | 0.75 |
| BMI WHO z scores | 1 | 222 | 2.39 (1.58, 3.20) | 0.00 | 222 | 1.32 (0.74, 1.90) | 0.00 |
|  | 2 | 166 | 2.09 (0.96, 3.22) | 0.00 | 166 | 1.40 (0.59, 2.21) | 0.00 |

| Exposure | Model | Systolic Blood Pressure (mmHg)  at adolescent visit | | | Diastolic Blood Pressure (mmHg)  at adolescent visit | | |
| --- | --- | --- | --- | --- | --- | --- | --- |
|  |  | **n** | **β (95% CI)** | **P** | **n** | **β (95% CI)** | **P** |
| **FEMALES** |  |  |  |  |  |  |  |
| Height (cm) | 1 | 227 | 0.35 (0.09, 0.61) | 0.01 | 227 | 0.13 (-0.03, 0.31) | 0.12 |
|  | 2 | 187 | 0.30 (-0.02, 0.63) | 0.07 | 187 | 0.12 (-0.09, 0.35) | 0.25 |
| Leg length (cm) | 1 | 223 | -0.07 (-0.41, 0.27) | 0.68 | 223 | -0.15 (-0.39, 0.07) | 0.17 |
| Trunk length (cm) | 1 | 223 | 0.80 (0.44, 1.17) | 0.00 | 223 | 0.47 (0.22, 0.71) | 0.00 |
|  | 2 | 185 | 0.76 (0.29, 1.22) | 0.00 | 185 | 0.47 (0.15, 0.79) | 0.00 |
|  | 3 | 185 | 0.75 (0.28, 1.22) | 0.00 | 185 | 0.46 (0.14, 0.78) | 0.00 |
| Leg-to-trunk ratio | 1 | 223 | -29.67 (-49.5, -9.7) | 0.00 | 223 | 0.47 (0.22, 0.71) | 0.00 |
|  | 2 | 185 | -30.02 (-55.1, -4.8) | 0.02 | 185 | -22.33 (-39.4, -5.2) | 0.01 |
| BMI WHO z scores | 1 | 227 | 2.05 (1.12, 2.98) | 0.00 | 227 | 1.27 (0.65, 1.90) | 0.00 |
|  | 2 | 187 | 2.39 (1.26, 3.53) | 0.00 | 187 | 1.57 (0.80, 2.35) | 0.00 |

**Model 1:** age

**Model 2:** age, place of residence, birth length, birth weight for gestational age z score, gestational age, smoking, and alcohol use

**Model 3:** age, place of residence, birth length, birth weight for gestational age z score, gestational age, smoking, alcohol use, and other component of current height (leg length for trunk length and vice versa)
