# Supplementary material for: Association of anthropometric measures and cardiovascular risk factors in children and adolescents: Findings from the Aboriginal Birth Cohort study
Source: PLoS One. 2018 Jun 21;13(6):e0199280. doi: 10.1371/journal.pone.0199280 (PMC6013209; doi:10.1371/journal.pone.0199280)
Supplement: S3 Table — (DOCX) [file pone.0199280.s003.docx]

Supplementary Table 3: Associations between anthropometric measures at birth and childhood with cholesterol, HDL-c and LDL-c measured at the childhood and adolescent visit for males and females using the complete case data

| Exposure | Model | Cholesterol (mmol/L)  at childhood visit | | | Cholesterol (mmol/L)  at adolescent visit | | |
| --- | --- | --- | --- | --- | --- | --- | --- |
|  |  | n | β (95% CI) | P | n | β (95% CI) | P |
| **MALES** |  |  |  |  |  |  |  |
| **Birth** |  |  |  |  |  |  |  |
| Birth weight (kg) | 1 | 281 | 0.07 (-0.06, 0.21) | 0.29 | 223 | 0.10 (-0.07, 0.29) | 0.25 |
| Birth length (cm) | 1 | 276 | 0.00 (-0.03, 0.03) | 0.99 | 220 | 0.00 (-0.03, 0.04) | 0.86 |
| **Childhood** |  |  |  |  |  |  |  |
| Height (cm) | 1 | 280 | 0.00 (-0.01, 0.01) | 0.53 | 212 | 0.01 (0.00, 0.02) | 0.04 |
|  | 2 | 222 | -0.00 (-0.01, 0.01) | 0.89 | 180 | 0.01 (-0.01, 0.02) | 0.34 |
| Leg length (cm) | 1 | 212 | 0.01 (-0.01, 0.02) | 0.33 | 167 | 0.02 (-0.00, 0.04) | 0.09 |
| Trunk length (cm) | 1 | 213 | 0.00 (-0.01, 0.02) | 0.52 | 168 | 0.02 (-0.00, 0.04) | 0.07 |
| Leg-to-trunk ratio | 1 | 212 | 0.14 (-0.72, 1.01) | 0.33 | 167 | 0.14 (-0.97, 1.26) | 0.80 |
| BMI WHO z scores | 1 | 280 | 0.10 (0.04, 0.16) | 0.00 | 212 | 0.20 (0.13, 0.27) | 0.00 |
|  | 2 | 222 | 0.10 (0.02, 0.17) | 0.01 | 180 | 0.20 (0.12, 0.29) | 0.00 |

| Exposure | Model | Cholesterol (mmol/L)  at childhood visit | | | Cholesterol (mmol/L)  at adolescent visit | | |
| --- | --- | --- | --- | --- | --- | --- | --- |
|  |  | n | β (95% CI) | P | n | β (95% CI) | P |
| **FEMALES** |  |  |  |  |  |  |  |
| **Birth** |  |  |  |  |  |  |  |
| Birth weight (kg) | 1 | 249 | 0.10 (-0.05, 0.26) | 0.19 | 225 | 0.17 (-0.01, 0.37) | 0.07 |
| Birth length (cm) | 1 | 245 | 0.02 (-0.01, 0.05) | 0.14 | 221 | 0.04 (-0.00, 0.08) | 0.05 |
|  | 2 | 219 | 0.03 (-0.02, 0.10) | 0.25 | 207 | 0.03 (-0.04, 0.11) | 0.41 |
| **Childhood** |  |  |  |  |  |  |  |
| Height (cm) | 1 | 249 | -0.00 (-0.01, 0.00) | 0.71 | 214 | -0.00 (-0.01, 0.02) | 0.65 |
| Leg length (cm) | 1 | 191 | -0.01(-0.03, 0.01) | 0.34 | 169 | -0.01 (-0.03, 0.01) | 0.42 |
| Trunk length (cm) | 1 | 191 | 0.01 (-0.01, 0.03) | 0.15 | 169 | 0.08 (-0.01, 0.04) | 0.14 |
| Leg-to-trunk ratio | 1 | 191 | -0.66 (-1.60, 0.26) | 0.16 | 169 | -0.74 (-1.90, 0.41) | 0.20 |
| BMI WHO z scores | 1 | 249 | 0.06 (-0.00, 0.12) | 0.08 | 214 | 0.09 (0.00, 0.18) | 0.04 |
|  | 2 | 219 | 0.05 (-0.02, 0.13) | 0.21 | 196 | 0.02 (-0.06, 0.12) | 0.53 |

**Model 1:** age

**Model 2:** age, place of residence, birth length, birth weight for gestational age z score, gestational age, and pubertal status (pubertal status adjusted only in childhood visit)

| Exposure | Model | HDL-c (mmol/L)  at childhood visit | | | HDL-c (mmol/L)  at adolescent visit | | |
| --- | --- | --- | --- | --- | --- | --- | --- |
|  |  | n | β (95% CI) | P | n | β (95% CI) | P |
| **MALES** |  |  |  |  |  |  |  |
| **Birth** |  |  |  |  |  |  |  |
| Birth weight (kg) | 1 | 280 | 0.01 (-0.04, 0.07) | 0.53 | 223 | -0.01 (-0.06, 0.04) | 0.69 |
| Birth length (cm) | 1 | 275 | 0.00 (-0.01, 0.01) | 0.48 | 220 | -0.00 (-0.01, 0.01) | 0.92 |
| **Childhood** |  |  |  |  |  |  |  |
| Height (cm) | 1 | 279 | 0.00 (-0.00, 0.01) | 0.16 | 212 | -0.00 (-0.01, 0.00) | 0.37 |
|  | 2 | 221 | 0.00 (-0.01, 0.00) | 0.95 | 180 | -0.00 (-0.00, 0.00) | 0.46 |
| Leg length (cm) | 1 | 211 | 0.00 (-0.00, 0.01) | 0.23 | 167 | -0.00 (-0.01, 0.01) | 0.62 |
| Trunk length (cm) | 1 | 212 | 0.00 (-0.00, 0.01) | 0.47 | 168 | -0.00 (-0.01, 0.00) | 0.48 |
| Leg-to-trunk ratio | 1 | 211 | 0.01 (-0.36, 0.37) | 0.97 | 167 | -0.01 (-0.32, 0.30) | 0.95 |
| BMI WHO z scores | 1 | 279 | -0.00 (-0.02, 0.02) | 0.83 | 212 | -0.02 (-0.04, -0.01) | 0.01 |
|  | 2 | 221 | -0.03 (-0.05, -0.00) | 0.04 | 180 | -0.03 (-0.06, -0.01) | 0.01 |

| Exposure | Model | HDL-c (mmol/L)  at childhood visit | | | HDL-c (mmol/L)  at adolescent visit | | |
| --- | --- | --- | --- | --- | --- | --- | --- |
|  |  | n | β (95% CI) | P | n | β (95% CI) | P |
| **FEMALES** |  |  |  |  |  |  |  |
| **Birth** |  |  |  |  |  |  |  |
| Birth weight (kg) | 1 | 248 | 0.00 (-0.05, 0.06) | 0.97 | 225 | 0.04 (-0.01, 0.09) | 0.12 |
| Birth length (cm) | 1 | 244 | 0.00 (-0.01, 0.01) | 0.74 | 221 | 0.01 (-0.00, 0.02) | 0.09 |
|  | 2 | 218 | 0.01 (-0.01, 0.03) | 0.44 | 207 | 0.01 (-0.01, 0.03) | 0.40 |
| **Childhood** |  |  |  |  |  |  |  |
| Height (cm) | 1 | 248 | 0.00 (-0.00, 0.00) | 0.69 | 214 | -0.00 (-0.00, 0.00) | 0.48 |
| Leg length (cm) | 1 | 190 | -0.00 (-0.01, 0.01) | 0.58 | 169 | -0.00 (-0.01, 0.01) | 0.74 |
| Trunk length (cm) | 1 | 190 | 0.00 (-0.00, 0.01) | 0.36 | 169 | 0.00 (-0.01, 0.01) | 0.59 |
| Leg-to-trunk ratio | 1 | 190 | -0.07 (-0.42, 0.26) | 0.64 | 169 | 0.00 (-0.33, 0.34) | 0.98 |
| BMI WHO z scores | 1 | 248 | -0.01 (-0.03, 0.01) | 0.44 | 214 | -0.02 (-0.05, -0.00) | 0.05 |
|  | 2 | 218 | -0.04 (-0.06, -0.00) | 0.02 | 196 | -0.04 (-0.07, -0.01) | 0.00 |

**Model 1:** age

**Model 2:** age, place of residence, birth length, birth weight for gestational age z score, gestational age, and pubertal status (pubertal status adjusted only in childhood visit)

| Exposure | Model | LDL-c (mmol/L)  at childhood visit | | | LDL-c (mmol/L)  at adolescent visit | | |
| --- | --- | --- | --- | --- | --- | --- | --- |
|  |  | n | β (95% CI) | P | n | β (95% CI) | P |
| **MALES** |  |  |  |  |  |  |  |
| **Birth** |  |  |  |  |  |  |  |
| Birth weight (kg) | 1 | 280 | 0.03 (-0.08, 0.16) | 0.56 | 223 | 0.07 (-0.08, 0.23) | 0.36 |
| Birth length (cm) | 1 | 275 | -0.01 (-0.03, 0.18) | 0.51 | 220 | -0.00 (-0.03, 0.03) | 0.91 |
| **Childhood** |  |  |  |  |  |  |  |
| Height (cm) | 1 | 279 | -0.00 (-0.00, 0.01) | 0.99 | 212 | 0.01 (-0.00, 0.02) | 0.06 |
|  | 2 | 221 | -0.00 (-0.01, 0.01) | 0.67 | 180 | 0.01 (-0.01, 0.02) | 0.38 |
| Leg length (cm) | 1 | 211 | 0.01 (-0.01, 0.02) | 0.43 | 167 | 0.01 (-0.01, 0.03) | 0.22 |
| Trunk length (cm) | 1 | 212 | 0.00 (-0.01, 0.02) | 0.74 | 168 | 0.02 (0.01, 0.04) | 0.01 |
| Leg-to-trunk ratio | 1 | 211 | 0.18 (-0.58, 0.95) | 0.64 | 167 | -0.42 (-1.33, 0.48) | 0.35 |
| BMI WHO z scores | 1 | 279 | 0.07 (0.02, 0.12) | 0.01 | 212 | 0.15 (0.09, 0.21) | 0.00 |
|  | 2 | 221 | 0.08 (0.01, 0.14) | 0.02 | 180 | 0.16 (0.09, 0.23) | 0.00 |

| Exposure | Model | LDL-c (mmol/L)  at childhood visit | | | LDL-c (mmol/L)  at adolescent visit | | |
| --- | --- | --- | --- | --- | --- | --- | --- |
|  |  | n | β (95% CI) | P | n | β (95% CI) | P |
| **FEMALES** |  |  |  |  |  |  |  |
| **Birth** |  |  |  |  |  |  |  |
| Birth weight (kg) | 1 | 247 | 0.04 (-0.10, 0.18) | 0.58 | 225 | 0.15 (-0.01, 0.31) | 0.06 |
| Birth length (cm) | 1 | 243 | 0.01 (-0.01, 0.04) | 0.37 | 221 | 0.03 (-0.00, 0.06) | 0.08 |
|  | 2 | 217 | 0.03 (-0.02, 0.10) | 0.23 | 207 | 0.01 (-0.04, 0.08) | 0.59 |
| **Childhood** |  |  |  |  |  |  |  |
| Height (cm) | 1 | 247 | -0.01 (-0.01, 0.00) | 0.26 | 214 | -0.00 (-0.01, 0.01) | 0.92 |
| Leg length (cm) | 1 | 189 | -0.01 (-0.03, 0.01) | 0.30 | 169 | -0.01 (-0.03, 0.01) | 0.33 |
| Trunk length (cm) | 1 | 189 | 0.01 (-0.01, 0.02) | 0.37 | 169 | 0.01 (-0.01, 0.03) | 0.30 |
| Leg-to-trunk ratio | 1 | 189 | -0.58 (-1.43, 0.26) | 0.17 | 169 | -0.57 (-1.54, 0.40) | 0.24 |
| BMI WHO z scores | 1 | 247 | 0.00 (-0.05, 0.06) | 0.86 | 214 | 0.08 (0.01, 0.16) | 0.02 |
|  | 2 | 217 | 0.00 (-0.07, 0.08) | 0.91 | 196 | 0.03 (-0.03, 0.11) | 0.32 |

**Model 1:** age

**Model 2:** age, place of residence, birth length, birth weight for gestational age z score, gestational age, and pubertal status (pubertal status adjusted only in childhood visit)
