# Supplementary material for: Association of anthropometric measures and cardiovascular risk factors in children and adolescents: Findings from the Aboriginal Birth Cohort study
Source: PLoS One. 2018 Jun 21;13(6):e0199280. doi: 10.1371/journal.pone.0199280 (PMC6013209; doi:10.1371/journal.pone.0199280)
Supplement: S4 Table — (DOCX) [file pone.0199280.s004.docx]

Supplementary Table 4: Associations between anthropometric measures at adolescence with cholesterol, HDL-c and LDL-c measured at the adolescent visit for males and females using the complete-case data

| Exposure | Model | Cholesterol (mmHg)  at adolescent visit | | | HDL-c (mmHg)  at adolescent visit | | | LDL-c (mmHg)  at adolescent visit | | |
| --- | --- | --- | --- | --- | --- | --- | --- | --- | --- | --- |
|  |  | n | β (95% CI) | P | n | β (95% CI) | P | n | β (95% CI) | P |
| **MALES** |  |  |  |  |  |  |  |  |  |  |
| Height (cm) | 1 | 222 | 0.00 (-0.01, 0.02) | 0.70 | 222 | -0.00 (-0.01, 0.00) | 0.68 | 222 | 0.00 (-0.01, 0.01) | 0.78 |
| Leg length (cm) | 1 | 215 | -0.02 (-0.05, -0.00) | 0.03 | 215 | 0.00 (-0.01, 0.01) | 0.91 | 215 | -0.02 (-0.04, 0.00) | 0.06 |
|  | 2 | 161 | -0.03 (-0.06, -0.00) | 0.03 | 161 | 0.00 (-0.01, 0.01) | 0.53 | 161 | -0.03 (-0.05, -0.00) | 0.04 |
|  | 3 | 161 | -0.03 (-0.07, -0.00) | 0.03 | 161 | 0.00 (-0.01, 0.01) | 0.50 | 161 | -0.03 (-0.05, -0.00) | 0.04 |
| Trunk length (cm) | 1 | 215 | 0.03 (0.01, 0.06) | 0.01 | 215 | -0.00 (-0.01, 0.00) | 0.32 | 215 | 0.02 (0.00, 0.04) | 0.05 |
|  | 2 | 161 | 0.02 (-0.02, 0.05) | 0.30 | 161 | -0.01 (-0.02, 0.00) | 0.21 | 161 | 0.01 (-0.02, 0.04) | 0.37 |
| Leg-to-trunk ratio | 1 | 215 | -2.87(-4.46, -1.27) | 0.00 | 215 | 0.24 (-0.23, 0.72) | 0.32 | 215 | -2.03 (-3.42, -0.65) | 0.00 |
|  | 2 | 161 | -2.48 (-4.60, -0.35) | 0.02 | 161 | 0.44 (-0.16, 1.05) | 0.15 | 161 | -2.02 (-3.87, -0.18) | 0.03 |
| BMI WHO z scores | 1 | 222 | 0.21 (0.15, 0.27) | 0.00 | 222 | -0.02 (-0.04, -0.01) | 0.01 | 222 | 0.13 (0.08, 0.19) | 0.00 |
|  | 2 | 164 | 0.27 (0.18, 0.35) | 0.00 | 164 | -0.04 (-0.07, -0.01) | 0.00 | 164 | 0.17 (0.09, 0.25) | 0.00 |

| Exposure | Model | Cholesterol (mmHg)  at adolescent visit | | | HDL-c (mmHg)  at adolescent visit | | | LDL-c (mmHg)  at adolescent visit | | |
| --- | --- | --- | --- | --- | --- | --- | --- | --- | --- | --- |
|  |  | n | β (95% CI) | P | n | β (95% CI) | P | n | β (95% CI) | P |
| **FEMALES** |  |  |  |  |  |  |  |  |  |  |
| Height (cm) | 1 | 225 | 0.00 (-0.01, 0.02) | 0.67 | 225 | 0.00 (-0.01, 0.01) | 0.87 | 225 | -0.00 (-0.01, 0.01) | 0.96 |
| Leg length (cm) | 1 | 220 | -0.01 (-0.04, 0.01) | 0.20 | 220 | -0.00 (-0.01, 0.01) | 0.88 | 220 | -0.02 (-0.04, 0.01) | 0.12 |
| Trunk length (cm) | 1 | 220 | 0.03 (-0.00, 0.05) | 0.06 | 220 | 0.00 (-0.07, 0.01) | 0.76 | 220 | 0.02 (-0.00, 0.05) | 0.11 |
| Leg-to-trunk ratio | 1 | 220 | -1.66 (-3.24, -0.08) | 0.04 | 220 | -0.09 (-0.55, 0.36) | 0.68 | 220 | -1.40 (-2.74, -0.07) | 0.04 |
|  | 2 | 184 | -0.71 (-2.50, 1.07) | 0.43 | 184 | 0.38 (-0.16, 0.92) | 0.17 | 184 | -0.94 (-2.43, 0.55) | 0.21 |
| BMI WHO z scores | 1 | 225 | 0.11 (0.03, 0.18) | 0.00 | 225 | -0.04 (-0.06, -0.02) | 0.00 | 225 | 0.08 (0.02, 0.14) | 0.01 |
|  | 2 | 187 | 0.11 (0.03, 0.19) | 0.01 | 187 | -0.05 (-0.07, -0.03) | 0.00 | 187 | 0.08 (0.01, 0.14) | 0.02 |

**Model 1:** age

**Model 2:** age, place of residence, birth length, birth weight for gestational age z score, gestational age, smoking and alcohol use

**Model 3:** age, place of residence, birth length, birth weight for gestational age z score, gestational age, smoking, alcohol use, and other component of current height (leg length for trunk length and vice versa)
