# Supplementary material for: Association of anthropometric measures and cardiovascular risk factors in children and adolescents: Findings from the Aboriginal Birth Cohort study
Source: PLoS One. 2018 Jun 21;13(6):e0199280. doi: 10.1371/journal.pone.0199280 (PMC6013209; doi:10.1371/journal.pone.0199280)
Supplement: S5 Table — (DOCX) [file pone.0199280.s005.docx]

Supplementary Table 5: Characteristics of ABC study participants – number who responded to each variable

|  | Total | Males | Females |
| --- | --- | --- | --- |
| **Birth** |  |  |  |
| Birth length (cm), n, mean (SD) | 649 48.8 (2.9) | 342 49.3 (3.0) | 307 48.3 (2.8) |
| Birth weight (kilograms), n, mean (SD) | 661 3.04 (0.63) | 347 3.13 (0.64) | 314 2.95 (0.59) |
| Gestational age (weeks), n, mean (SD) | 587 38.8 (1.7) | 301 38.8 (1.7) | 286 38.7 (1.7) |
| Place of residence, n (%) | 660 | 347 | 313 |
| Urban | 20.9% | 21.9% | 19.8% |
| Remote/Very Remote | 70.5% | 69.7% | 71.2% |
| Other | 8.6% | 8.4% | 8.9% |
| **Childhood visit (~11 years)** |  |  |  |
| Age at wave 2 (years), n, mean (SD) | 566 11.4 (1.1) | 301 11.5 (1.1) | 265 11.3 (1.1) |
| Place of residence, n (%) | 566 | 301 | 265 |
| Urban | 17.8% | 19.3% | 16.2% |
| Remote/Very Remote | 72.6% | 70.7% | 74.7% |
| Other | 9.5% | 9.9% | 9.0% |
| Pubertal status, n (%) | 535 | 281 | 254 |
| Pre-pubertal | 51.5% | 66.5% | 35.0% |
| Pubertal | 48.4% | 33.5% | 65.0% |
| Height (cm), n, mean (SD) | 565 143.7 (10.5) | 300 143.6 (10.4) | 265 143.9 (10.7) |
| Leg length (cm), n, mean (SD) | 426 71.7 (6.4) | 225 71.8 (6.7) | 201 71.7 (6.0) |
| Trunk length (cm), n, mean (SD) | 427 71.7 (6.5) | 226 71.5 (6.7) | 201 71.8 (6.3) |
| Weight (kg), n, mean (SD) | 565 35.9 (11.8) | 300 35.4 (11.9) | 265 36.3 (11.8) |
| Total cholesterol, n, mmol/L, mean (SD) | 530 4.0 (0.7) | 281 4.1 (0.7) | 249 3.9 (0.7) |
| HDL-c, mmol/L, n, mean (SD) | 528 1.2 (0.3) | 280 1.2 (0.3) | 248 1.1 (0.2) |
| LDL-c, mmol/L, n, mean (SD) | 527 2.2 (0.6) | 280 2.3 (0.7) | 247 2.2 (0.6) |
| Systolic blood pressure (mmHg), n, mean (SD) | 553 107.4 (10.2) | 292 107.5 (9.9) | 261 107.3 (10.6) |
| Diastolic blood pressure (mmHg), n, mean (SD) | 553 68.0 (7.1) | 292 67.4 (7.0) | 261 68.6 (7.2) |
| **Adolescent visit (~18 years)** |  |  |  |
| Age at wave 3 (years), n, mean (SD) | 469 18.2 (1.0) | 234 18.3 (1.0) | 235 18.1 (1.1) |
| Place of residence, n (%) | 478 | 238 | 240 |
| Urban | 19.5% | 21.8% | 17.1% |
| Remote/Very Remote | 66.7% | 64.2% | 69.2% |
| Other | 13.8% | 13.9% | 13.7% |
| Do you smoke tobacco/cigarettes? | 407 | 197 | 210 |
| No | 30.7% | 28.9% | 32.4% |
| Used to smoke | 1.5% | 2.0% | 0.9% |
| Smoke sometimes | 67.8% | 69.0% | 66.7% |
| Alcohol use | 437 | 213 | 224 |
| Never or only tried it once | 54.5% | 44.6% | 63.8% |
| Used to use it but not anymore | 3.2% | 2.8% | 3.6% |
| Sometimes | 42.3% | 52.6% | 32.6% |
| Height (cm), n, mean (SD) | 468 167.6 (8.7) | 234 173.6 (6.9) | 234 161.6 (5.7) |
| Leg length (cm), n, mean (SD) | 448 83.7 (5.7) | 221 87.3 (4.5) | 227 80.1 (4.4) |
| Trunk length (cm), n, mean (SD) | 448 83.8 (4.8) | 221 86.2 (4.6) | 227 81.6 (4.0) |
| Weight (kg), n, mean (SD) | 468 60.8 (19.0) | 233 66.1 (21.1) | 235 55.5 (14.9) |
| Total cholesterol, mmol/L, n, mean (SD) | 448 4.0 (0.8) | 223 4.1 (0.8) | 225 4.0 (0.8) |
| HDL-c, mmol/L, n, mean (SD) | 448 1.0 (0.2) | 223 1.0 (0.2) | 225 1.0 (0.2) |
| LDL-c, mmol/L, n, mean (SD) | 448 2.3 (0.7) | 223 2.4 (0.7) | 225 2.3 (0.7) |
| Systolic blood pressure (mmHg), n, mean (SD) | 451 109.4 (11.8) | 224 112.5 (11.3) | 227 106.4 (11.5) |
| Diastolic blood pressure (mmHg), n, mean (SD) | 451 68.7 (7.8) | 224 68.9 (7.8) | 227 65.6 (7.7) |
