# Supplementary material for: Association of anthropometric measures and cardiovascular risk factors in children and adolescents: Findings from the Aboriginal Birth Cohort study
Source: PLoS One. 2018 Jun 21;13(6):e0199280. doi: 10.1371/journal.pone.0199280 (PMC6013209; doi:10.1371/journal.pone.0199280)
Supplement: S6 Table — (DOCX) [file pone.0199280.s006.docx]

Supplementary Table 6: Associations between anthropometric measures at birth and childhood with cholesterol, HDL-c and LDL-c measured at the childhood and adolescent visit for males and females using the multiply imputed data

| Exposure | Model | Cholesterol (mmol/L) at childhood visit | | Cholesterol (mmol/L) at adolescent visit | | HDL (mmol/L)  at childhood visit | | HDL (mmol/L)  at adolescent visit | | LDL (mmol/L)  at childhood visit | | LDL (mmol/L)  at adolescent visit | |
| --- | --- | --- | --- | --- | --- | --- | --- | --- | --- | --- | --- | --- | --- |
|  |  | β  (95% CI) | P | β  (95% CI) | P | β  (95% CI) | P | β  (95% CI) | P | β  (95% CI) | P | β  (95% CI) | P |
| **MALES** |  |  |  |  |  |  |  |  |  |  |  |  |  |
| **Birth** |  |  |  |  |  |  |  |  |  |  |  |  |  |
| Birth weight (kg) | 1 | 0.07  (-0.06, 0.21) | 0.28 | 0.13  (-0.04, 0.31) | 0.14 | 0.01  (-0.04, 0.07) | 0.59 | 0.01  (-0.04, 0.05) | 0.71 | 0.03  (-0.09, 0.16) | 0.55 | 0.09  (-0.05, 0.25) | 0.20 |
| Birth length (cm) | 1 | 0.01  (-0.02, 0.03) | 0.67 | 0.01  (-0.02, 0.05) | 0.45 | 0.00  (-0.01, 0.01) | 0.62 | 0.00  (-0.00, 0.01) | 0.37 | -0.00  (-0.03, 0.02) | 0.89 | 0.01  (-0.02, 0.03) | 0.62 |
| **Childhood** |  |  |  |  |  |  |  |  |  |  |  |  |  |
| Height (cm) | 1 | 0.01  (-0.01, 0.02) | 0.32 | 0.01  (-0.00, 0.02) | 0.09 | 0.00  (-0.00, 0.01) | 0.11 | -0.00  (-0.00, 0.00) | 0.59 | 0.00  (-0.00, 0.01) | 0.87 | 0.01  (-0.00, 0.02) | 0.16 |
| Leg length (cm) | 1 | 0.01  (-0.01, 0.02) | 0.38 | 0.01  (-0.01, 0.02) | 0.43 | 0.00  (-0.00, 0.01) | 0.39 | -0.00  (-0.00, 0.00) | 0.32 | 0.00  (-0.01, 0.01) | 0.73 | 0.00  (-0.01, 0.01) | 0.82 |
| Trunk length (cm) | 1 | 0.00  (-0.01, 0.01) | 0.52 | 0.01  (-0.00, 0.03) | 0.23 | 0.00  (-0.00, 0.01) | 0.21 | 0.00  (-0.01, 0.00) | 0.88 | 0.00  (-0.01, 0.01) | 0.91 | 0.01  (-0.00, 0.03) | 0.15 |
| Leg-to-trunk ratio | 1 | 0.11  (-0.60, 0.83) | 0.75 | -0.12  (-1.15, 0.89) | 0.80 | -0.06  (-0.41, 0.27) | 0.69 | -0.11  (-0.40, 0.18) | 0.45 | 0.11  (-0.55, 0.78) | 0.74 | -0.47  (-1.29, 0.34) | 0.25 |
| BMI WHO z scores | 1 | 0.07  (0.02, 0.13) | 0.01 | 0.13  (0.06, 0.19) | 0.00 | 0.00  (-0.02, 0.02) | 0.87 | -0.01  (-0.03, 0.01) | 0.23 | 0.05  (0.00, 0.10) | 0.03 | 0.10 (0.04, 0.15) | 0.00 |
|  | 2 | 0.06  (-0.00, 0.11) | 0.06 | 0.12  (0.05, 0.19) | 0.00 | -0.02  (-0.04, 0.00) | 0.10 | -0.02  (-0.04, 0.01) | 0.07 | 0.05  (-0.01, 0.10) | 0.10 | 0.09 (0.03, 0.15) | 0.00 |

| Exposure | Model | Cholesterol (mmol/L) at childhood visit | | Cholesterol (mmol/L) at adolescent visit | | HDL (mmol/L)  at childhood visit | | HDL (mmol/L)  at adolescent visit | | LDL (mmol/L)  at childhood visit | | LDL (mmol/L)  at adolescent visit | |
| --- | --- | --- | --- | --- | --- | --- | --- | --- | --- | --- | --- | --- | --- |
|  |  | β  (95% CI) | P | β  (95% CI) | P | β  (95% CI) | P | β  (95% CI) | P | β  (95% CI) | P | β  (95% CI) | P |
| **FEMALES** |  |  |  |  |  |  |  |  |  |  |  |  |  |
| **Birth** |  |  |  |  |  |  |  |  |  |  |  |  |  |
| Birth weight (kg) | 1 | 0.10  (-0.04, 0.25) | 0.18 | 0.13  (-0.04, 0.31) | 0.12 | 0.01  (-0.04, 0.06) | 0.74 | 0.04  (-0.01, 0.10) | 0.12 | 0.03  (-0.09, 0.17) | 0.58 | 0.11  (-0.03, 0.26) | 0.13 |
| Birth length (cm) | 1 | 0.02  (-0.01, 0.05) | 0.18 | 0.03  (-0.00, 0.07) | 0.09 | 0.00  (-0.01, 0.01) | 0.65 | 0.01  (-0.00, 0.02) | 0.05 | 0.01  (-0.02, 0.03) | 0.52 | 0.02  (-0.00, 0.05) | 0.14 |
|  | 2 | 0.02  (-0.03, 0.09) | 0.40 | 0.03  (-0.05, 0.10) | 0.48 | 0.01  (-0.01, 0.03) | 0.63 | 0.01  (-0.01, 0.03) | 0.26 | 0.01  (-0.04, 0.07) | 0.64 | 0.01  (-0.05, 0.07) | 0.79 |
| **Childhood** |  |  |  |  |  |  |  |  |  |  |  |  |  |
| Height (cm) | 1 | 0.00  (-0.01, 0.02) | 0.46 | 0.00  (-0.01, 0.01) | 0.69 | 0.00  (-0.00, 0.01) | 0.33 | 0.00  (-0.00, 0.00) | 0.90 | -0.00  (-0.01, 0.01) | 0.95 | 0.00  (-0.01, 0.01) | 0.91 |
| Leg length (cm) | 1 | -0.01  (-0.02, 0.01) | 0.60 | -0.01  (-0.02, 0.01) | 0.54 | -0.00  (-0.00, 0.01) | 0.62 | -0.00  (-0.00, 0.00) | 0.27 | -0.00  (-0.02, 0.01) | 0.60 | -0.01  (-0.02, 0.01) | 0.40 |
| Trunk length (cm) | 1 | 0.01  (-0.00, 0.02) | 0.22 | 0.01  (-0.01, 0.03) | 0.15 | 0.00  (-0.00, 0.01) | 0.25 | 0.00  (-0.00, 0.00) | 0.30 | 0.00  (-0.01, 0.02) | 0.56 | 0.00  (-0.00, 0.02) | 0.35 |
| Leg-to-trunk ratio | 1 | -0.43  (-1.25, 0.38) | 0.29 | -0.63  (-1.62, 0.36) | 0.21 | -0.11  (-0.43, 0.19) | 0.45 | -0.15  (-0.45, 0.14) | 0.31 | -0.32  (-1.09, 0.44) | 0.40 | -0.50  (-1.35, 0.34) | 0.24 |
| BMI WHO z scores | 1 | 0.04  (-0.01, 0.10) | 0.15 | 0.06  (-0.02, 0.13) | 0.17 | 0.00  (-0.02, 0.03) | 0.97 | -0.00  (-0.02, 0.01) | 0.67 | 0.00  (-0.04, 0.06) | 0.83 | 0.05  (-0.01, 0.11) | 0.13 |

**Model 1:** age

**Model 2:** age, place of residence, birth length, birth weight for gestational age z score, gestational age, and pubertal status (pubertal status adjusted only in childhood visit)
