# Supplementary material for: Association of anthropometric measures and cardiovascular risk factors in children and adolescents: Findings from the Aboriginal Birth Cohort study
Source: PLoS One. 2018 Jun 21;13(6):e0199280. doi: 10.1371/journal.pone.0199280 (PMC6013209; doi:10.1371/journal.pone.0199280)
Supplement: S7 Table — (DOCX) [file pone.0199280.s007.docx]

Supplementary Table 7: Associations between anthropometric measures at adolescence with cholesterol, HDL-c and LDL-c measured at the adolescent visit for males and females using the multiply imputed data

| Exposure | Model | Cholesterol (mmol/L)  at adolescent visit | | HDL-c (mmol/L)  at adolescent visit | | LDL-c (mmol/L)  at adolescent visit | |
| --- | --- | --- | --- | --- | --- | --- | --- |
| **MALES** |  | β (95% CI) | P | β (95% CI) | P | β (95% CI) | P |
| Height (cm) | 1 | 0.01  (-0.01, 0.02) | 0.55 | 0.00  (-0.00, 0.01) | 0.70 | 0.00  (-0.01, 0.02) | 0.68 |
| Leg length (cm) | 1 | -0.02  (-0.05, -0.00) | 0.05 | 0.00  (-0.00, 0.01) | 0.86 | 0.00  (-0.01, 0.01) | 0.82 |
|  | 2 | -0.02  (-0.05, -0.00) | 0.04 | 0.00  (-0.01, 0.00) | 0.90 | -0.02  (-0.04, -0.00) | 0.04 |
|  | 3 | -0.02  (-0.05, -0.00) | 0.04 | 0.00  (-0.01, 0.01) | 0.89 | -0.02  (-0.04, -0.00) | 0.04 |
| Trunk length (cm) | 1 | 0.03  (0.00, 0.06) | 0.02 | -0.00  (-0.00, 0.00) | 0.73 | 0.02  (-0.00, 0.04) | 0.07 |
|  | 2 | 0.02  (-0.01, 0.06) | 0.19 | -0.01  (-0.02, 0.00) | 0.16 | -0.01  (-0.01, 0.04) | 0.34 |
| Leg-to-trunk ratio | 1 | -2.65  (-4.40, -0.91) | 0.00 | 0.12  (-0.35, 0.60) | 0.60 | -1.95  (-3.44, -0.47) | 0.01 |
|  | 2 | -2.12  (-3.92, -0.33) | 0.02 | 0.29  (-0.21, 0.80) | 0.24 | -1.55  (-3.08, -0.02) | 0.05 |
| BMI WHO z scores | 1 | 0.13  (0.07, 0.19) | 0.00 | -0.02  (-0.03,-0.00) | 0.04 | 0.09  (0.04, 0.14) | 0.00 |
|  | 2 | 0.12  (0.05, 0.19) | 0.00 | -0.02  (-0.04, 0.01) | 0.01 | 0.08  (-0.02, 0.14) | 0.01 |

| Exposure | Model | Cholesterol (mmol/L)  at adolescent visit | | HDL-c (mmol/L)  at adolescent visit | | LDL-c (mmol/L)  at adolescent visit | |
| --- | --- | --- | --- | --- | --- | --- | --- |
| **FEMALES** |  | β (95% CI) | P | β (95% CI) | P | β (95% CI) | P |
| Height (cm) | 1 | 0.00  (-0.00, 0.01) | 0.38 | 0.00  (-0.00, 0.01) | 0.87 | -0.00  (-0.01, 0.01) | 0.94 |
| Leg length (cm) | 1 | -0.02  (-0.04, 0.00) | 0.15 | 0.00  (-0.00, 0.00) | 0.77 | -0.01  (-0.02, 0.01) | 0.40 |
| Trunk length (cm) | 1 | 0.02  (-0.00, 0.05) | 0.08 | 0.00  (-0.00, 0.01) | 0.57 | 0.01  (-0.01, 0.04) | 0.19 |
| Leg-to-trunk ratio | 1 | -1.64  (-3.15, -0.13) | 0.03 | -0.05  (-0.51, 0.40) | 0.81 | -1.34  (-2.62, -0.06) | 0.04 |
|  | 2 | -0.73  (-2.34, 0.88) | 0.37 | 0.33  (-0.17, 0.85) | 0.18 | -0.72  (-2.07, 0.61) | 0.28 |
| BMI WHO z scores | 1 | 0.08  (0.01, 0.15) | 0.03 | -0.02  (-0.04, -0.01) | 0.01 | 0.06  (-0.00, 0.12) | 0.05 |
|  | 2 | 0.05  (-0.01, 0.13) | 0.13 | -0.04  (-0.05, -0.02) | 0.000 | 0.04  (-0.01, 0.10) | 0.17 |

**Model 1:** age

**Model 2:** age, place of residence, birth length, birth weight for gestational age z score, gestational age, smoking and alcohol use

**Model 3:** age, place of residence, birth length, birth weight for gestational age z score, gestational age, smoking, alcohol use, and other component of current height (leg length for trunk length and vice versa)
